# How do Internal Medicine Residents from Different Backgrounds Make Subspecialty Career Choices? A Qualitative Analysis

**DOI:** 10.1007/s11606-025-09967-9

**Published:** 2025-11-21

**Authors:** Blythe Butler, Ana I. Velazquez, Evelin Trejo, Laura A. Huppert, Gerald Hsu, Jennifer M. Babik, Lekshmi Santhosh

**Affiliations:** 1 Department of Medicine, Division of Pulmonary, Critical Care, Allergy & Sleep Medicine, University of California San Francisco, San Francisco, CA USA; 2 Department of Medicine, Division of Hematology/Oncology at Zuckerberg San Francisco General, University of California San Francisco, San Francisco, CA USA; 3 Department of Medicine, Division of Hematology/Oncology, University of California, San Francisco, San Francisco, CA USA; 4 Department of Medicine, Division of Hematology/Oncology at the San Francisco Veterans Affairs Medical Center, University of California San Francisco, San Francisco, CA USA; 5 Department of Medicine, Division of Infectious Diseases, University of California San Francisco, San Francisco, CA USA

## Abstract

**Purpose:**

Medical career decisions are complex. Gender and racial disparities remain among internal medicine (IM) subspecialty fellows, particularly in procedural subspecialties. This qualitative study aims to thematically explore factors that influence IM resident subspecialty career choice, with a focus on factors specific to women and UIM trainees to better understand existing disparities. This is the first study to explore IM residents own stated reasons to subspecialize into various fields.

**Methods:**

We conducted virtual focus groups with 37 IM residents from multiple institutions, organized by affinity groups based on self-identifying as UIM and non-UIM race or ethnicity. Thematic analysis using inductive coding was used to analyze the data and identify themes applying the Systems Theory Framework (STF) of career development. Key themes were divided into social or contextual factors, individual factors, or uncontrollable factors.

**Results:**

Study participants were 45.9% women (*n* = 17) and 51.4% self-identified as UIM (*n* = 19). In this sample of graduating IM residents who have matched into subspeciality fellowships, contextual factors emerged as the most influential to their career choices. The main contributors to selecting a subspecialty were mentorship and role models, positive experiences and exposure to the field, work-life balance, enjoyment of clinical and/or procedural work, and financial compensation. UIM residents identified their communities and their personal identities (i.e., race, ethnicity, culture) as influential to their career choices. Both women and UIM residents more frequently described the importance of representation and belonging in their career decisions compared to their non-UIM and male counterparts. Family planning emerged as an important factor for women.

**Conclusions:**

Our findings lend insights into how academic institutions, medicine subspecialty societies, training programs, and faculty at large can engage trainees in their field. We propose ways to improve the recruitment and retention of women and UIM trainees into the IM subspecialty fields.

**Supplementary Information:**

The online version contains supplementary material available at 10.1007/s11606-025-09967-9.

## INTRODUCTION

Medical career decisions are inherently complex processes driven by countless variables and experiences that vary in importance from person to person, such as experiences during clinical training,^1^ racial and ethnic background,^2^ gender,^3^ level of debt,^4^ and mentorship.^5^ The influence of individual experiences and larger societal level variables on career choice are best understood in context of the individual making the decision. In the USA, more than 25% of postgraduate training positions are in internal medicine (IM),^6^ and therefore there is significant interest in factors that affect IM trainees career choice. Moreover, stated career choices before and during IM residency training have low predictability toward final career choice, indicating experiences during training play a significant role.^7^

It is also well established that a diverse physician workforce can improve the physician productivity and performance,^8^ the quality of healthcare delivered,^8^ health disparities,^9^ and the clinical learning environment.^10^ Despite this, women and underrepresented in medicine (UIM) physicians remain underrepresented across all internal medicine (IM) subspecialties.^11^ Enrollment data from the Accreditation Council for Graduate Medical Education (ACGME) showed the percentage of women fellows decreased from 1991 to 2016, from 33.3 to 23.6%.^12^ This trend is concordant with prior studies showing that men IM graduates were more likely to enter subspecialty training than women,^13^ and that women IM residents were more likely to report generalist career plans.^7,14^ A separate study revealed that while the overall percentage of UIM subspecialty fellows increased from 2006 to 2018, there was substantial variation by subspecialty.^11^ UIM fellows remain substantially underrepresented compared to the US population. These disparities are more pronounced within procedural subspecialties (i.e., cardiology, gastroenterology, and pulmonary/critical care).^11^ Moreover, in 2018, no subspecialties reflected the diversity of the US population, and more than half had lower percentages of UIM fellows than IM residencies. This is a useful benchmark, as residents are the direct pipeline for these fellowships,^11^ and UIM residents are entering all subspecialty fellowships at a disproportionately lower rate than their non-UIM counterparts.

Understanding factors that influence IM resident career choices broadly and women and UIM residents specifically is critical to understanding and addressing this leaky pipeline. Several other studies have described the dynamic nature of subspecialty career choice during IM residency training^7^and the current state of subspecialty fellowships.^11,15^ There are numerous quantitative studies documenting disparities in career choice, with methods such as examining NRMP/ERAS data sources^11^and ITE data.^16^ However, this is the first study to systematically use a qualitative approach to explore IM residents’ own stated reasons behind the choice to subspecialize into various fields—or not. Moreover, this unique study specifically recruited women and UIM residents to elicit these responses. This qualitative study aims to elucidate factors that influence IM resident subspecialty career choices with a particular focus on women and UIM medicine residents to attempt to better understand reasons behind the persistent gender and racial/ethnic disparities in representation across IM subspecialty fields.

## METHODS

### Study Design

We conducted a qualitative research study to investigate factors that influence subspecialty career choices among IM residents, with a particular focus on factors affecting UIM and women residents. To elicit a diversity of experiences and to explore the potential role of racial and ethnic identity on career choices, focus groups were organized by affinity groups based on self-identifying as UIM vs. non-UIM. This study was deemed exempt by the University of California, San Francisco Institutional Review Board. LS is an intensivist and hospitalist, ET is a PhD behavioral scientist with expertise in qualitative research, and AMV is a hematologist/oncologist. All authors have extensive workplace teaching experience, as well as formal training in Health Professions Education research, including qualitative methods.

We attempted to capture and analyze the complexity of these career decisions, adopting the Systems Theory Framework (STF) to guide our work.^17^ The STF is a framework of career development that visually depicts the interconnectedness of individual, social, and societal systems. This framework offers a means of understanding the wide range of influences that are likely to impact career choice and development, divided according to whether they occur within the individual, within the individual’s professional context, or within the individual’s broader environment. It has been used in prior studies analyzing career decision-making in different populations.^18^ STF allowed us to pay careful attention to themes emerging from residents’ perspectives on their subspecialty career choice and to contextualize themes that surfaced more frequently from specific groups, i.e., UIM and women residents. This was valuable given the current state of disparities in representation across all IM subspecialties.^11^

### Study Participants and Recruitment

Using convenience sampling, we recruited third-year IM residents or fourth-year chief residents from US-based, ACGME-accredited, IM residency programs who were applying to a medicine subspecialty fellowship. Participation was voluntary and participants were recruited via email (Appendix) and social media, leveraging the study team’s professional networks, site-specific residency mailing lists, and participant referrals, through a broad social media campaign and using list-serves to all residents at geographically diverse residency programs as well as leveraging professional connections. All focus groups were conducted in 2022 after the medicine subspecialty Fellowship Match Day (December 2021) to eliminate conflicts of interest and bias during the fellowship recruitment process. Participants were recruited and interviewed until thematic saturation was achieved.^19^

### Focus Groups

We conducted eight focus groups total (five UIM and three non-UIM affinity focus groups), with two to six medicine residents in each focus group. All participants provided electronic consent via Qualtrics (Qualtrics, Provo, UT) and verbal consent before study participation. Each participant completed a brief anonymous demographic survey. All focus groups were conducted virtually via Zoom (Zoom Video Communications, Inc., San Jose, CA),^20,21^ and were facilitated by one of three study investigators (L.S., A.I.V., L.A.H.) using an open-ended approach and semi-structured questions developed from a review of the literature on gender and racial disparities in medicine and career choice as well feminist theory, intersectionality, and professional identity formation theoretical frameworks.^22–24^ The focus group guide was pilot tested with participants and revised accordingly. Questions centered on exploring career path and goals, and influential factors to subspecialty choice, such as training and clinical experiences, extracurricular activities, mentorship and role models, professional environment, personal identities, belonging, culture, lifestyle, and financial considerations (Appendix). Focus groups lasted approximately 60 min and were recorded. Audio recordings were transcribed by a professional transcription service (Rev.com, Inc., Austin, TX), deidentified, and reviewed for accuracy against audio recordings prior to analysis. Each study participant received a $40 gift card as compensation for their time and effort.

### Data Analysis

Based on grounded theory, we used inductive coding to analyze the transcribed focus groups.^25,26^ Two investigators (E.T., A.I.V.) reviewed two transcripts selected at random and created an initial codebook to inductively create data-driven themes and codes.^27–29^ The study team (E.T., A.I.V., L.S., B.B.) reviewed and refined the codes, definitions, application of codes, and coding structure developing a final codebook. Using an updated codebook, two investigators (E.T., B.B.) then independently applied codes to all transcripts and documented potential themes in memos. Any coding discrepancies were reviewed by a third independent investigator (A.I.V.) and discussed as a group and resolved with consensus of the study team. The study team then reviewed and grouped similar codes into themes in an iterative process, and subsequently grouped the identified themes applying the STF of career development.^17,30,31^ The STF provides a comprehensive framework for understanding the wide range of factors at the individual-level (i.e., intrapersonal factors), social system (i.e., contextual factors), and environmental/societal systems (i.e., uncontrollable factors) that can influence career development, taking into account both content and process influences.^17,18^ Finally, we then reviewed the representative quotes within each theme and sub-theme to compare if each factor was equally cited across all IM residents or more prevalent amongst non-UIM, UIM, or women subgroups.

## RESULTS

**Figure 1:**
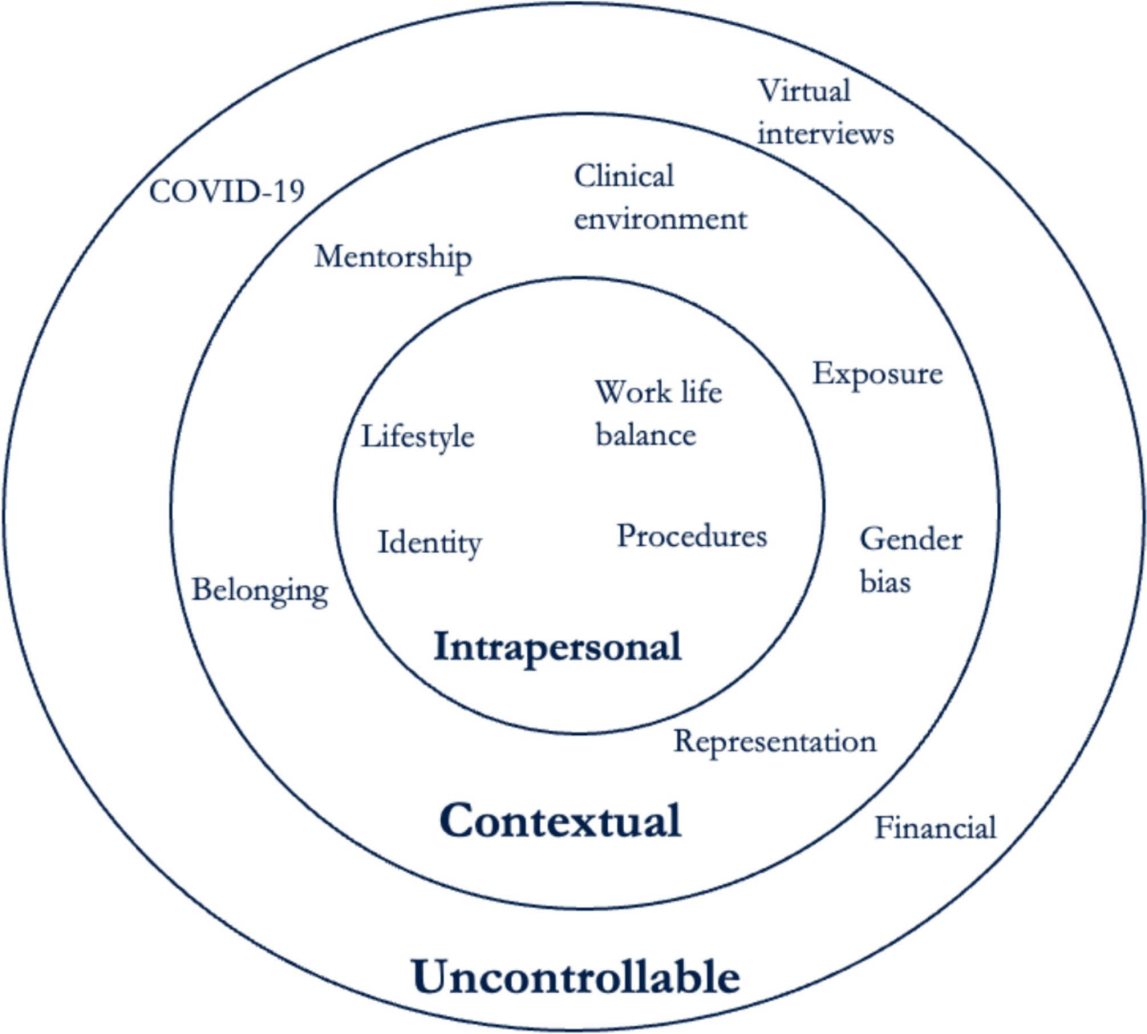
Adapted Systems Theory Framework (STF) with key themes identified during the focus groups with internal medicine residents influencing subspecialty career choice.

A total of 37 IM residents participated in eight focus groups, five UIM and three non-UIM affinity focus groups. Participants were 45.9% women (*n* = 17) and 51.4% self-identified as UIM (*n* = 19); 16 IM residency programs and 9 prospective subspecialty fellowships were represented (Table 1). Key themes identified in all focus groups were grouped into social and contextual factors, individual factors, and environmental or uncontrollable factors. These themes and subthemes are presented in the following sub-sections (Fig. 1). Social and contextual factors were the main factors described by residents as influential to their subspecialty choice, including *the importance of mentorship and role models, sense of belonging, representation in the field*, *community service*, *gender bias*, *microaggressions*, and *culture.* Residents also described influential individual factors, such as *identity*, *work-life balance and lifestyle*, and *type of clinical work* (e.g., inpatient versus outpatient setting, procedural work). Residents identified environmental or uncontrollable factors that influenced their specialty choices, like *immigration status*, *financial considerations*, and *the COVID-19 pandemic.* Given the disparities previously noted, we performed additional analysis of the differences in subthemes out of the UIM vs non-UIM focus groups as well as the different subthemes highlighted between by women residents. We found UIM resident’s responses more frequently reflected the subthemes of representation, sense of belonging, identity, and the importance of mentorship as compared to their non-UIM counterparts. Women resident’s responses more frequently reflected gender bias and work-life balance as compared to men.

**Table 1:** Internal Medicine Resident Participant Characteristics (*n* = 37)

Characteristic	*N* (%)
**Sex**	
Female Male	17 (45.9) 20 (54.1)
**Race/ethnicity**	
UIM Non-UIM	19 (51.4) 18 (48.6)
**Subspecialty choice**	
Addiction medicine Cardiology Endocrinology Gastroenterology Geriatrics/palliative care Hematology/oncology Infectious disease Nephrology Pulmonary/critical care	1 (2.7) 8 (21.6) 1 (2.7) 6 (16.2) 1 (2.7) 10 (27) 1 (2.7) 2 (5.4) 7 (19.0)
**Region**	
Midwest Northeast South West Other (US territory)	5 (13.5) 10 (27.0) 4 (10.8) 17 (46.0) 1 (2.7)
Residency programs* (*N*)	16

Fifteen participants (40.5%) belonged to one unique residency program; the other 15 residency programs had a range of participants of 1–3 per program

### Social and Contextual Factors

#### Importance of Mentorship and Role Models

Most IM residents identified early mentorship and research as drivers of their interest in a particular subspecialty. Identifying mentors and role models allowed residents to see a future career path in their subspecialty of interest.

“… a sub interest of mine all throughout was healthcare disparities and vulnerable populations and I wasn’t really sure how those two things fit together […] I always saw oncology research is so basic science heavy and clinical trials heavy, but [I] found mentors in residency that helped me to see a career pathway for me that [I] could study health disparities research in the oncology space.” [Focus Group (FG)1, non-UIM]

Further, both non-UIM and UIM residents discussed identifying mentors invested in their career gave them a sense of community and reassured their career choices:

(Referring to mentors) ”They were very interested in my career. That was one of the things that made me feel comfortable in choosing that career, because I felt that I had a community that would help me get to where I wanted to get.” [FG3, UIM]

#### Belonging and Shared Identities

UIM and non-UIM residents described looking for mentors that they could relate to as a constant conscious or subconscious process during training. Relating to mentors in different aspects of life, such as shared personal identities or common education background, influenced residents’ career choices and allowed them to imagine a long-term career in the field.

“…[I] have shared a lot of similarities with [my research mentors], whether it's race or growing up in a similar part of the country or going to the same med [school] […] In the process as I was seeking out mentors, whether consciously or subconsciously, I realized I wanted to be able to see myself […] in their shoes in 10, 20 years.” [FG8, non-UIM]

UIM residents specifically described the importance of representation and commonly shared challenges while identifying role models and mentors they could relate to. Notably, identifying mentors and faculty with shared identities in their particular fellowship program created a sense of belonging and reinforced the career choices of UIM residents:

“…initially […] it didn’t really feel like a good fit, but I did meet Black and Brown [doctors] that were gastroenterologists. And for me that’s when it did feel like it was going to be a good fit because I saw people that looked similar to me, they came from similar walks of lives as me.” [FG6, UIM]

UIM residents described the weight and negative impact of the lack of workforce diversity on their career choices:

“I still struggle with this, good fit for me. […] I don’t see myself still as a subspecialist. […] Part of it is not seeing a ton of women of color, people of color in the subspecialty. I think it helps when I do [to] reinforce like, oh, you do belong here, this is a good fit for you. These are your people. But it’s kind of few and far between.” [FG6, UIM]

Conversely, some UIM residents saw lack of mentorship and representation as an opportunity to diversify their subspecialties and to become mentors to future minority trainees:

“… I have not found within cardiology someone that I identify with and look up to […] not having the type of people that I was looking for within cardiology was another big motivation for me […], We need more Latinos in cardiology. […] I hope that in the future, I can be that one person that someone is looking to as a mentor. […] This is a field that needs people to diversify.” [FG3, UIM]

Residents also described personality traits as perceived factors that could influence subspecialty choice and their “fit” within a field. For example, some residents described wanting to avoid the competitive nature of certain subspecialties, or being told they were “too nice” to be in a particular subspecialty.

#### Gender Bias and Underrepresentation of Women

Women residents often described lack of gender diversity in their chosen subspecialty field, especially for procedural specialties, and how it affected their choice of fellowship program:

“It influenced my choice […] for fellowship, because there were [places] that I interviewed that had maybe one or two female faculty out of 30, 40, 50, and none on the selection committee, and maybe very few female fellows.” [FG5, non-UIM]

Further, women residents described interactions during training with faculty who questioned their ability to balance their chosen subspecialty work demands with stereotypical gender roles such as home keeping or childcare:

“…It’s actually ironically individuals outside the field that have questioned my choice, specifically based on my gender, and actually the fact that my husband is also a physician has influenced this as well because they ask, oh, well who’s going to stay home and take care of the kids? Does the ICU allow that? [FG7, non-UIM]

#### Serving the Community

UIM and non-UIM residents described relationships with patients as drivers of their clinical interest in a subspecialty field. UIM residents were more likely to cite the health disparities in their own their communities as influential. For example, a UIM participant described their lived experiences growing up in a marginalized community and how it impacted their commitment to serve their community:

“I don’t think necessarily my race or ethnicity played a role in that [referencing career choice] more than just how I grew up and where I grew up, and the disparities that I saw in my community […] I think it’s more broad than just being [Latinx group]. It’s more being [from] a community that’s marginalized and underserved.” [FG3, UIM].

UIM residents were also more likely to describe motivation to serve patients who shared their racial/ethnic background or language:

“I felt like as I progressed through my career and could see room for the provider to look like the patient, I felt very motivated.” [FG1, UIM].

### Individual Level Factors

#### Personal Identities

Personal identities were a major part of the decision-making process for most residents. Among UIM residents, race, ethnicity, and language were often mentioned as unique parts of their identity that sparked their interest in certain diseases and subspecialties:

"My identity played a role in me being more of a malignant hematology guy, […] I recognize that there aren’t many Black physicians in that space… my interest in multiple myeloma is really just me having an interest in disparities and people in African descent." [FG*3, UIM*] 

"Within Latinos, there’s a lot of cardiovascular disease, and I felt that I could make an impact. In the area of [state] where I’m from, […], I don’t know any cardiologist who’s a Spanish speaker that could contribute to the health needs of that community where I ultimately want to work at." [FG3, UIM] 

#### Family and Work-Life Balance

Many IM residents described the importance of being able to prioritize time with their family in a future career in their chosen subspecialty field:

“And so I felt like that part of the culture was really important to me, is being able to prioritize family, and prioritize other parts of my life, and feeling that support.” [FG3, UIM]

Many women residents described a perceived conflict with wanting to balance family planning and a demanding subspecialty. Some residents mentioned the importance of having mentors who modeled parenthood in medicine:

“… Three of the four of my research mentors have taken maternity leave while I've worked with them […] As a female in academic medicine, […] being able to see other people do that successfully was really helpful.” [FG7, non-UIM]

Achieving the desired work-life balance during and after training was described as important by most residents. When making decisions regarding their subspecialty, residents reflected on their future goals and their perceptions of faculty work-life balance, especially in procedural subspecialties:

“Seeing what it’s like to have a really heavy clinical workload, and potentially taking a step back and saying, "What other things do I want to fill my life?"[…] As I go on, there are a lot of other areas I could be much happier without the same work demands as [interventional cardiology].” [FG5, non-UIM]

Further, residents noted an important desire to preserve their own identity and prioritize aspects of their life outside of medicine:

“…Just being a doctor is not who I am. I prefer to be recognized as a friend, as a family person, and hopefully in the future as a husband of my future wife. That’s to me way more important than just saying, yeah, I’m the best cardiologist in the city. […] I do believe that in the end what really matters is just your relationships with your family and your friends. […] I do believe that having a life outside medicine is way more important than being a doctor.” [FG6, UIM]

#### Individual Preference Related to Clinical Work and Procedures

We found that all IM residents, regardless of gender or UIM status, considered their preference practice settings and procedural work. All residents also highlighted the importance of considering the diversity of work settings, practice types, and the ability to focus and develop expertise on a specific disease:

"I like the fact that in pulmonary critical care fellowship, you can have outpatient consult and also critical care experience. I really liked that kind of lifestyle. […] I figured out that pulmonary and critical fellowship has a little bit of everything." [FG4, UIM]

### Environmental and Uncontrollable Factors

#### COVID-19 and Virtual Interviews

The decrease of in-person experiences due to the COVID-19 pandemic and the transition to a virtual fellowship application process decreased interactions with faculty, diminished opportunities for residents to work in subspecialty clinics, and increased stress regarding the match process and the inability to visit other campuses and meet their faculty:

"I think the most stressful experience for me was when COVID started, the clinic stopped, so we couldn’t go to clinic. So, I was really stressed because I couldn’t get more clinical experience outpatient with my mentors." [FG*4, UIM*] 

#### Financial Considerations

Financial compensation was commonly mentioned when choosing a subspecialty. Nearly all interviewed residents discussed differences in compensation among subspecialties, the burden of deferred income while prolonging training, and their financial responsibilities with family as influential to their decision-making:

“… financial is one of the things that I would say would’ve been a negative factor for going to primary care, I also come from a poorer environment and both of my parents are getting near retirement” [FG1, UIM]

## DISCUSSION

This study examines factors influencing subspeciality career choices among IM residents by using a qualitative approach to analyze their own stated reasons behind these choices, with a particular focus on the unique perspective of women and UIM IM residents to better understand existing disparities. It is the first study to our knowledge to do so. Through a STF analysis, we were able to build a complex framework though which to understand the way in which IM residents placed emphasis on various individual, interpersonal and systems level factors that influenced their decision to pursue a subspecialty. We found that when choosing a subspecialty, all IM residents considered pragmatic factors, such as work-life balance, financial compensation, exposure to a field during training, and procedural nature of their chosen field.

These findings align with and build upon the current literature on factors influencing overall resident subspecialty choices. Kua and colleagues conducted a longitudinal single institution survey study evaluating resident career choices in Singapore and identified clinical exposure to a field, influences from peers and elders, and lifestyle choices as influential to residents’ subspecialty choices.^32^ In contrast, Yang et al.’s study showed that early exposure to subspeciality rotations did not predict final career choice for subspecialties.^7^

**Figure 2:**
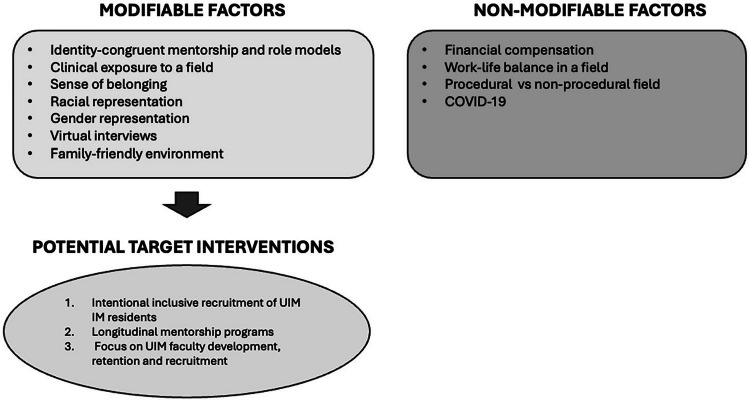
Modifiable and non-modifiable factors affecting UIM resident career choice.

As above, we noted distinct themes in the factors that influenced UIM residents’ subspecialty choice compared with their non-UIM counterparts (Fig. 2). From the influences identified, particularly from the subthemes that were more frequently identified amongst UIM and women residents, we propose actions targeted interventions at hospitals and subspecialty training programs. While all residents considered pragmatic factors as described, UIM residents were more likely to discuss the importance of their own cultural and racial identities. In contrast, non-UIM residents did not discuss race or racism in their groups. This may be in part to how the focus groups were structured (i.e., by self-identified UIM status) and is a potential limitation of our study. UIM residents were also much more likely to stress the importance of identity-concordant mentorship and racial representation. Additionally, UIM residents cited a lack of representation as something that diminished their sense of belonging and interest in pursuing a subspecialty. This aligns with findings by Agawu et al.,^3^ whose study highlighted that UIM medical students choose residency fields where they foresee acceptance and community. It is not surprising that trainees desire and seek out support in their racial, ethnic, and cultural identities while pursuing their careers. Together this offers a plausible explanation for the trends in racial and ethnic diversity in IM subspecialties, as described by Santhosh and Babik of our team.^11^ Thus, the lack of faculty reflecting the identities of UIM residents in these fields potentially negatively impacts the ability to recruit UIM residents.

Like with UIM residents, several themes emerged as concerns shared by self-identified women residents. They were more likely to discuss the challenge of balancing family planning and career, especially when considering procedural fields, consistent with the findings in Garibaldi et al.^33^ There were no instances of men discussing their gender or balancing family life as it pertained to career choice in our focus groups. Women residents also discussed having their career choices questioned and having to justify their choice of a “demanding” subspecialty. Gender disparities in IM subspecialties may persist and be reinforced as trainees perceive certain fields as less compatible with family life and encounter fewer female faculty role models.

The lessons from this study suggest specific recruitment and retention strategies for UIM residents in medicine to address the current leaky pipeline. First, UIM residents should be encouraged and intentionally recruited to apply to a diverse array of IM subspecialties. Gonzaga and others provide a framework for inclusive recruitment strategies in graduate medical education.^34^ This requires dedicated, longitudinal mentoring from diverse faculty who reflect the identities of UIM residents so that residents can identify role models in their field of interest. Hemal and group describe longitudinal mentoring programs and holistic review during recruitment for increasing UIM and women representation among surgical trainees, and similar strategies can likely be applied to IM subspecialty fields.^35^

Second, recruitment and retention of UIM fellows onto faculty should be prioritized. This faculty community can serve as mentors to IM residents, improve the sense of belonging amongst UIM trainees so that they can serve as mentors to IM residents, and improve patient care and UIM trainee clinical experience. Similar to GME trainees, longitudinal mentorship of UIM faculty can lead to successful recruitment, retention, career satisfaction, and improved patient care. While training, recruiting, and retaining UIM and female faculty at individual institutions in specialties where they are underrepresented is ideal, this is unlikely to happen immediately. Other “bridges” such as promoting and supporting access to national networks of UIM or female mentors in the field, providing training for non-UIM or non-female mentors to be more skilled in “mentoring across differences,” and creating networks of near peer mentors in intended specialties can be very impactful.

This study has multiple strengths. Utilizing the STF, which has been previously applied to academic medicine career choice,^32,36^ provided us with a structure to understand both individual and external factors associated with subspecialty choices and how these may influence residents decisions to pursue a fellowship.^30,31^ Additionally, the use of focus groups allowed for deeper exploration of this topic.^37^ Further, grouping residents in affinity groups strengthened the depth of discussion by promoting social connectivity, belonging, and diminishing power dynamics and stereotype threat, especially among participants with marginalized identities.^38–40^ This is also a potential confounder, as it is possible that participants’ responses may have been overly influenced by themes discussed by peers within their focus group.

Additional limitations of this study include the relatively small sample size and potential for sampling bias. Though we aimed for a broad, diverse, and inclusive sample, self-selection bias can impact and subsequently over-represent specific viewpoints of our participants. Although our sample included representation from a wide array of IM subspecialty fields, cardiology, hematology/oncology, and pulmonary/critical care were more heavily represented. Most of the interviewees were residents from large academic programs who self-elected to participate and were recruited either by mailing lists, referrals, or online recruitment. The study did not include perspectives of IM residents who considered pursuing a subspecialty path but ultimately decided not to. Understanding the reasons for these decisions would add important understanding of additional factors that affect career decision making. Additionally, 40% of the participants were from a single residency program at the same institution as the researchers conducting the study. Therefore, it is possible that participants’ responses may have reflected biased viewpoints or exaggerated the strength of a particular influence, thus limiting the generalizability of our results. Finally, we did not report the results by institution or geographic area to maintain the anonymity of our participants.

In summary, our study highlights the role of mentorship and role models in the career decisions of medicine residents as well as the specific importance of contextual factors such as belonging, representation, and community alignment for UIM and women residents. These findings lend insights into how academic institutions, medicine subspecialty societies, training programs, and faculty at large can engage trainees and improve the recruitment and retention of women and UIM trainees into the IM subspecialty fields. While IM residents may weigh specific factors and their individual lived experiences differently in their career choices, we believe focusing on the mentorship experiences of UIM and women residents is the most impactful and pressing issue to increase diversity in the IM subspecialties.^31^

## Supplementary Information

Below is the link to the electronic supplementary material.

ESM 1 (DOCX 32.2 KB)

## Data Availability

All data are publicly available upon request.
