# Supplementary material for: How do Internal Medicine Residents from Different Backgrounds Make Subspecialty Career Choices? A Qualitative Analysis
Source: J Gen Intern Med. 2025 Nov 21;41(3):653–62. doi: 10.1007/s11606-025-09967-9 (PMC12960857; doi:10.1007/s11606-025-09967-9)
Supplement: Supplementary file 1 — (DOCX 32.2 KB) [file 11606_2025_9967_MOESM1_ESM.docx]

**Appendix**

**Email promo**

**Invite to Participate in Subspecialty Career Choice Study**

Dear [X],

We are inviting you to take part in a research study led by Dr. Lekshmi Santhosh focused on evaluating disparities in subspecialty career choice among internal medicine residents.

Your participation in the study would consist of completing a short online demographics questionnaire that should take less than 5 minutes and participation in one 60–90 minute focus group. If you participate in the focus group, you will receive a $50 Amazon gift card as compensation for your time and effort.

Participation is completely voluntary, and the decision to participate or not in this study will not impact future training, job, or educational opportunities.

***If you are interested in participating, please complete the screening questionnaire below.*** If you are eligible to participate, our study team will contact you within the next 72 hours.

**Screening Questionnaire Link:**

Your input is invaluable!

Thank you for considering,

Consent and Screening Questionnaire

**UNIVERSITY OF CALIFORNIA, SAN FRANCISCO**

**Information Sheet: Focus Groups**

**Study Title: A Mixed-Methods Analysis of Gender & Racial Disparities in Subspecialty Career Choice**

We would like to invite you to participate in a research study evaluating disparities in subspecialty career choice among internal medicine residents.

The purpose of this study is to elucidate the factors that influence internal medicine resident subspecialty career choice and evaluate differences in experience and priorities when making career choices by gender and racial/ethnic group.

You are being invited to consider participating in a focus group as part of this study. We ask you review the following facts and consider completing the screening questionnaire below. If you are eligible, after completion of the screening questionnaire study staff will contact you within 72 hours for consent, completion of a short demographic survey, and scheduling of the focus group.

- - We are asking you to participate as you are a current internal medicine trainee or are currently in a fellowship program.
  - Participation in this study is voluntary.
  - The decision to participate in this study will not impact future training, job, or educational opportunities nor will answers be shared with your current or future schools, or employers in an identifiable way.
  - Your participation in the study would consist of completing a short online demographics questionnaire (estimated completion time less than 5 minutes) and participation in one 60-90 minute focus group.
  - We aim to enroll 30-45 individuals with a goal of 50% who self-identify as underrepresented in medicine (UIM) and 50% women.
  - Focus groups may be held over zoom or, if needed, in-person.
  - When possible, focus groups will be held by affinity groups (UIM vs non-UIM and by gender) and will consist of 8-12 participants.
  - If you participate in the focus group you will receive a $50 Amazon gift card as compensation for your time and effort.
  - We will make a digital recording of the focus groups. This information will be used together with your survey responses. We will do our best to make sure that your personal information will be kept private and secure. After the interview, our research team will type into a computer a transcription of what has been recorded and will remove any mention of names. The written transcript of the digital recording and the recordings themselves will be identified by a code number only. After all the interviews for the study have been analyzed, the recording will then be destroyed.
  - Any identifiable information will be stored separately from study data and will not be included in the analysis.
  - There is minimal to no risk for study participation.
  - The raw responses/data will be seen by members of the research team for aggregation, analysis, and creation of generalizable knowledge.
  - This study is sponsored by UCSF’s Academy of Medical Educators.
  - If you have any questions, you may contact the study PI, Dr. Lekshmi Santhosh.
  - If you have questions or concerns about your rights as a research participant, you can call the UCSF Institutional Review Board at 415-476-1814.

Thank you for your interest in participating in this study!

Lekshmi Santhosh, MD

University of California, San Francisco

Email: [lekshmi.santhosh@ucsf.edu](mailto:lekshmi.santhosh@ucsf.edu)

If you are interested in participating in this study, please complete the following screening questionnaire. If you are eligible, after completion of the screening questionnaire study staff will contact you within 72 hours for consent, completion of a short demographic survey, and scheduling of the focus group.

Consent to complete the screening questionnaire is implied by selecting “Yes” in the form below. If you do not wish to be considered for participation in this study, please select “No” or select “X” in the top corner of your browser.

I agree to complete the screening questionnaire:

☐ Yes

☐ No

**SCREENING QUESTIONNAIRE**

1. **Please select your current year of graduate medical training:**

☐ PGY-1

☐ PGY-2

☐ PGY-3

☐ PGY-4

☐ PGY-5 or more

☐ Decline to state

1. **Please select your current level of training program:**

☐ Internal Medicine

☐ 4^th^ year Chief Resident

☐ Subspecialty Fellowship training

☐ 1-year fellowship training (i.e. hepatology, heart failure, bone marrow transplant, occupational medicine, etc.)

☐ Addiction Medicine, General Internal Medicine, or Hospital Medicine Fellowship

☐ Research fellowship

☐ Other: _____________

☐ Decline to state

1. **In what region of the country do you currently live/practice?**

☐ Northeast

☐ Midwest

☐ South

☐ West

☐ Other

☐ Decline to state

1. **Do you self-identify with a racial or ethnic group historically considered as underrepresented in medicine?**

*Underrepreented in medicine include those who self-identify as Black or African American, Latinx or Hispanic, Native Hawaiian or other Pacific Islander, Native American, American Indian, Alaska Native, Filipino, Hmong, or Vietnamese*.

☐ Yes

☐ No

☐ Other: _________

☐ Decline to state

1. **What is your gender identity?**

☐ Male

☐ Female

☐ Transgender man / Transman

☐ Transgender woman / Transwoman

☐ Genderqueer / Gender nonconforming

☐ Additional identity (fill in) ________________

☐ Decline to state

1. **Name: _______________________________**
2. **Preferred email address: ___________________**
3. **Preferred phone number: ___________________**
4. **What is your preferred contact method?**

☐ Email

☐ Phone

**Thank you!**

**If you are eligible for participation, we will contact you within 72 hours to inform you of your eligibility and next steps.**

If you have any questions, please contact our study team

Focus group interview guide

**UNIVERSITY OF CALIFORNIA, SAN FRANCISCO**

**FOCUS GROUP INTERVIEW GUIDE**

**Study Title: A Mixed-Methods Analysis of Gender & Racial Disparities in Subspecialty Career Choice**

Introduction:

Hello, my name is **____** and I am the (***role in study***) in this study. I will be facilitating our discussion today. (**if a Note Taker is present: This is . She/he/they will be taking notes and recording our conversation**.)

Thank you for volunteering to participate this study. The purpose of this study is to elucidate the factors that influence internal medicine resident subspecialty career choice and evaluate differences in experience and priorities when making career choices by gender and racial/ethnic group. During this focus group, you will answer open-ended questions about how you make choices regarding your medical specialty selection.

Because we want to make sure that we accurately capture all of your ideas, (***I vs. our note taker***) will be taking notes during our conversation and we will record our discussion.

Now, we will go over some ground rules for today:

- This is an informal discussion.
- We are interested in all your ideas, comments, and suggestions.
- There are no right or wrong answers. All comments, both positive and negative, are welcomed.
- Feel free to ask any questions as they come up.
- Your opinion is important to us, and we want you to feel comfortable in saying what you really think. Please speak one at a time so that we can listen to your opinions.
- If you disagree with another person’s opinion, we would like to hear your opinion, but please be respectful of those who disagree with you.
- To ensure everyone feels comfortable expressing their ideas at this discussion, we ask that you agree not to share the contents of the discussion or information about other focus group participants with anyone outside this setting. However, we cannot guarantee that each participant will keep the discussions private.
- The information you share will be kept confidential.
- We will refer to each other by first names. Your names will not be used in the notes or in any reports. No identifying information will be included in any publication or other dissemination of our work.
- If possible, please silence your phones to minimize interruptions during the focus group.
- (If on Zoom) If possible, please join this focus group in a private space and we ask you please mute yourself while you are not speaking.
- Being in this study is optional. Please tell us researcher if you do not want to participate.
- The decision to participate in this study will not impact future training, job, or educational opportunities nor will answers be shared with your current or future training programs, or employers in an identifiable way.
- As a reminder, our focus group today will likely last approximately 60-90 minutes. I will record the conversation, and it will be transcribed. The recording will be deleted after the anonymized transcript is complete. Members of the research team for this project will review interview transcripts and code them for themes.
- Questions? Please contact Lekshmi Santosh at [lekshmi.santosh@ucsf.edu](mailto:lekshmi.santosh@ucsf.edu). If you have questions or concerns about your rights as a research participant, you can call the UCSF Institutional Review Board at 415-476-1814.

**Do I have your verbal consent to proceed?**

Thank you. I will now begin recording our conversation. Please allow me a moment to get the recording started. **[*Start recording and dictate date, time, and focus group number*.]**

[Instructions for facilitator: These questions do not have to be read verbatim. They are topics to be used as a guide for steering the conversation.]

Great. Now we’ll get started. Does everyone know each other?

First, I’d like us to talk about your career path and goals.

1. Tell me about your current career goals and path.
   - Are you planning to pursue further subspecialty training?
2. How did you decide on that specialty and what was the decision process like?
   - When did you make your decision?
   - Was this an easy or difficult decision? Why?

Now let’s talk about why did you select this career path and what may have influenced your choice.

1. Please tell me why did you select that specialty of choice?
   - What things did you like?
   - Anything, you don’t like?
2. Did your experience with internal medicine residency affect that choice, and if so, how?

- *Probe:* What are some itples of situations or interactions that helped you decide on your current career goals/path?
- *Probe:* How do you think that your clinical experiences and exposure in your program affect your choices?

1. How, if at all, did your medical school training impact your choices?
2. What other factors did you take into account when deciding your career goals/path?
3. How do you think your experiences during the COVID-19 pandemic have shaped or affected your career choices?
   - Has is reinforced your choices?
   - Has it changed your interests? And how?

Now I would like to focus on people who may have influenced your career path.

1. Are there persons in your environment who shaped your career aspirations?
   - *Probe*: What are some examples of role models, mentors, patients, family, or friends who shaped your decision making?
2. To what degree and how did your identities affect your career choices?
   - How do you believe that your gender identity affected your choice?
   - How do you believe that your ethnicity affected your choice?
   - *Probe*: Did diversity/gender of faculty and fellows play a role in your decision making?
   - *Probe*: Did the perceived diversity of patients in X specialty play a role in your decision making?
3. To what degree and how did the “culture” and “personality” of a specialty affect your career choices?
   - *Probe:* Do you believe that identifying or not-identifying with faculty or fellows affected your decision making?
   - *Probe:* How does faculty work-life balance and satisfaction play a role on your decision making?
4. Do you think your specialty of choice provides an environment that is respectful of unique aspects of your identity (i.e. Race, gender identity, sexual orientation, etc.)?
   - How do you believe that experiencing bias towards one or more of your identities has affected your career choices?
   - *Probe:* How could it be more supportive and/or inclusive with regard to these parts of your identity?

**[*Closing remarks*]**

Thank you very much for taking the time to participate in this study. We may have follow-up questions after this focus group, would it be ok if we contacted you with further questions?

**[*End recording*]**
